# Application of a revised model for coping with advanced cancer to qualitatively explore lung cancer survivors’ experiences of ongoing physical effects, novel treatments, uncertainty, and coping

**DOI:** 10.1007/s11764-023-01417-x

**Published:** 2023-07-27

**Authors:** Rebekah Laidsaar-Powell, Phyllis Butow, Bernadette Bea Brown, Kimberley Mander, Jane Young, Emily Stone, Venessa Chin, Emily Banks, Chloe Yi Shing Lim, Nicole M Rankin

**Affiliations:** 1 Centre for Medical Psychology and Evidence-Based Decision-Making (CeMPED), School of Psychology, Faculty of Science, The University of Sydney, Sydney, NSW Australia; 2 NHMRC Clinical Trials Centre, Faculty of Medicine and Health, The University of Sydney, Sydney, NSW Australia; 3 Sydney School of Public Health, Faculty of Medicine and Health, The University of Sydney, Sydney, NSW Australia; 4 The Daffodil Centre, The University of Sydney, a joint venture With Cancer Council, Sydney, NSW Australia; 5 Department of Thoracic Medicine and Lung Transplantation, St Vincent’s Hospital Sydney, Sydney, Australia; 6 School of Clinical Medicine, UNSW, Sydney, Australia; 7 The Kinghorn Cancer Centre, 370 Victoria Street, Darlinghurst, Sydney, NSW 2010 Australia; 8 The Garvan Institute of Medical Research, Sydney, NSW Australia; 9 National Centre for Epidemiology and Population Health, Australian National University, Canberra, ACT Australia; 10 Centre for Health Policy, Melbourne School of Population and Global Health, The University of Melbourne, Melbourne, Australia

**Keywords:** Lung cancer, Survivorship, Qualitative, Immunotherapy, Coping and adjustment

## Abstract

**Purpose:**

Lung cancer remains underrepresented in cancer survivorship research. This study aimed to understand survivors’ physical/psychological challenges, experiences of immunotherapy (IO) and targeted therapy (TT), and psychological adjustment through application of the Roberts et al. (2017) advanced cancer adaptation of Folkman and Greer’s appraisal and coping model.

**Methods:**

Adults 6–24 months post-initial treatment completion were recruited via an Australian cohort study. Participant demographic, clinical, quality of life, and distress data were obtained through the cohort database. Qualitative interviews were conducted and analyzed using Framework methods. Roberts et al. (2017)’s model informed data interpretation and presentation.

**Results:**

Twenty interviews were conducted (10 females; average age 69 years). Participants’ diagnostic stages varied (stage I = 2, stage II = 4, stage III = 8, stage IV = 6); most had received IO/TT (*n* = 14) and were on average 17 months (range 10–24) post-diagnosis. Three themes were identified and mapped to the Roberts’ framework: (1) *Ongoing illness events:* most participants reported functioning well despite ongoing physical effects. Those on IO/TT reported side effects; some were unexpected/serious. (2) *Adjusting to life with lung cancer:* most expressed hope for the future while simultaneously preparing for disease progression. Those receiving IO/TT experienced uncertainty given limited survival information. (3) *Learning to live with lung cancer:* participants described emotion, problem, and meaning based on coping strategies.

**Conclusions:**

Findings may guide development of supportive care resources/interventions focused on uncertainty, IO/TT communication and decision-making, and coping.

**Implications for Cancer Survivors:**

Many people with lung cancer are living well with their ongoing illness. Despite challenges, many survivors are adapting to issues as they arise and are maintaining a sense of hope and optimism.

**Supplementary Information:**

The online version contains supplementary material available at 10.1007/s11764-023-01417-x.

## Introduction

Globally, lung cancer is the leading cause of cancer-related death and the second most diagnosed cancer [29]. Lung cancers are often diagnosed at an advanced stage, and the 5-year survival rate is poor, ranging from 10 to 20% across most countries [29]. However, many individuals with lung cancer are living longer due to earlier detection and improved medical treatments [2], such as immunotherapy (IO) and targeted systemic therapies (TT) [12]. Despite improving survivorship trajectories, both lung cancer and its treatments can lead to ongoing physical, functional, and psychosocial challenges. Indeed, lung cancer survivors report greater limitations to physical functioning and psychological distress and poorer quality of life (QoL) than the general population and survivors of other cancers [13]. Lung cancer survivors’ quality of life and psychological distress have been found to remain significantly poorer than the general population at 5 years post-surgery [21].

People living with lung cancer remain underrepresented in survivorship research [32], particularly qualitative research that explores experiences of individuals living with cancer for many years post-treatment. Indeed, our group’s meta-review of qualitative systematic reviews of cancer survivorship found very few reviews focusing on, or including, lung cancer survivors [15]. Furthermore, most qualitative studies conducted to date were published prior to widespread availability of novel therapies, particularly IO [7, 24], or have focused on specific survivorship issues such as stigma [6] or online support [33].

A handful of recent studies have focused on lung cancer survivors who have received novel therapies; these suggest such survivors may have unique and complex needs. A 2021 Australian study of 20 individuals with metastatic non-small cell lung cancer (NSCLC) treated with IO/TT identified experiences of chronic treatment-related toxicities, psychological concerns about uncertainty, and need for practical support and tailored information [14]. Another brief qualitative report on the experiences of metastatic lung cancer survivors receiving TT identified key informational and supportive care needs, including for help to navigate the persistent uncertainty related to ongoing treatment [20]. While these studies provide much needed insight into those people living with lung cancer, the brevity of results means more comprehensive exploration of the psychological experience of living longer-term with lung cancer is urgently needed.

Recent conceptual advancements can provide additional insights into the psychological experience of living with advanced and/or poor prognosis cancers, such as lung cancer. Roberts et al. (2017) revised the Folkman and Greer [8] model of appraisal and coping to accommodate the experience of advanced cancer as a fluctuating and chronic condition. Roberts’ framework posits that living with advanced cancer is a process of experiencing multiple fluctuating disease *events* (rather than a single acute illness episode). People with advanced cancer draw on *personal characteristics* which inform *appraisals* of their illness and repeatedly reframe and redevelop coping strategies to utilize in response to these fluctuating events. However, no studies to date have described application of this framework to lung cancer survivors’ experiences.

## Aim

This paper aims to describe the psychological experience of living with and potentially beyond lung cancer (6–24 months post-diagnosis), as seen through the lens of Roberts et al. (2017)’s revised model for coping with advanced cancer. Specifically, this paper reports on ongoing physical and psychological challenges, uncertainty, coping strategies, and experiences of and attitudes towards novel therapies such as IO/TT.

## Methods

This study is part of a larger qualitative project that also explored lung cancer survivors’ healthcare experiences, relationships with family and friends, and employment and financial concerns, which will be reported elsewhere. The study was conducted in accordance with the consolidated criteria for reporting qualitative research (COREQ) checklist for reporting qualitative research [30] (see Supplementary File B). Ethical approval for this project was authorized by Sydney Local Health District Lead Human Research Ethics Committee (RPA Zone) under protocol number X16-0447.

### Participants

Participants were recruited through their ongoing participation in a large Australian lung cancer clinical cohort study (Embedding Research and Evidence in Cancer Healthcare—EnRICH Program) [28]. All patients aged 18 years and over, presenting to defined clinical sites for the diagnosis or treatment of a new primary lung cancer, or first progressive disease, local recurrence, or new metastasis, between June 2017 and October 2021, are included in the EnRICH cohort. EnRICH participants who had previously consented to involvement in ongoing research and matched purposive selection parameters were approached to participate in the current study. The eligibility criteria for this qualitative study were age ≥ 18 years, 6–24 months post-completion of initial active treatment (e.g., lung resection, chemotherapy, radiotherapy), not receiving end-of-life care, and well enough and with adequate English language skills to complete the study requirements. Participants who were currently receiving ongoing IO/TT or maintenance chemotherapy were considered eligible to participate if they were between 6 and 24 months post-diagnosis.

Purposive sampling was utilized to ensure diversity across key participant characteristics and to allow exploration of differences/similarities across key issues. Key characteristics were informed by the evidence base, decided upon by all co-authors, and included early and advanced disease stages, receipt of IO/TT, and those with higher/lower quality of life.

### Recruitment

An EnRICH project officer (KM) phoned eligible EnRICH participants who met purposive sampling parameters to introduce the study and invite participation. Interested patients were contacted by the study manager (RL-P) to provide more detailed information. An information sheet and consent form were emailed/posted, and upon return, a telephone interview was arranged. Interviews continued until thematic saturation (no new themes identified after three consecutive interviews) was reached [18].

### Data collection

Participant demographic, clinical, and patient-reported outcome (PRO) data was obtained from the existing EnRICH cohort study database.

#### Demographic data

Included age, sex, country of birth, education, occupation, income, remoteness of residence, and marital status.

#### Clinical data

Included date of diagnosis, tumor stage, histological type, treatment modality and dates, number of comorbidities, and smoking status.

#### Patient-reported outcomes (PRO)

Included overall quality of life (EORTC-QLQ-C30) and distress (NCCN Distress Thermometer). The EORTC-QLQ-C30 [1] is a validated 30-item cancer-specific measure of quality of life. Transformed scores range from 0 to 100, with a higher score representing a better level of functioning. The Distress Thermometer is scored between 0 and 10 where higher scores indicate greater distress; scores of ≥ 5 indicate clinical levels of distress [25]. PROs were obtained via the EnRICH cohort longitudinal study which assesses QoL and distress among participants at diagnosis, 3, 6, and 12 months post-diagnosis and annually thereafter, with participants’ most recent scores being reported in this study.

#### Qualitative interviews

The semi-structured interview schedule was developed by three authors based on the literature and their expertise in psycho-oncology, qualitative methods, and lung cancer management (RL-P, PB, NR) and reviewed/revised by authors who have expertise in the clinical care of people diagnosed with lung cancer and research (health services research and epidemiology) (BB, KM, JY, ES, VC, EB, CL). Interviews explored the experience of lung cancer from diagnosis through treatment and then post-treatment with a focus on the physical, psychological, social, and lifestyle changes and challenges of living with lung cancer (see Supplementary File A for sample interview questions). Each participant completed the semi-structured interview via telephone, which was conducted by a female, PhD-qualified experienced qualitative researcher (RL-P). Prior to commencing interviews, RL-P reflected upon and documented personal beliefs/experiences, potential biases, and assumptions relevant to the research questions. Post-interview reflections were noted after each interview. This reflexive material was consulted throughout data analysis and interpretation. Interviews lasted on average 54 min (range 27–97 min). Audio recordings and verbatim transcripts of interviews comprise the raw data.

### Data analysis

Quantitative demographic, clinical, and PRO data were managed using summary statistics in Microsoft Excel 2016. Qualitative data were analyzed using the Framework approach. Framework methods are a matrix-based analytic method used to conduct thematic analysis. Framework methods are commonly used in applied health research and provide a structured and standardized approach to qualitative analysis. Framework methods facilitate comparisons within and between cases and allow for sub-group analysis according to key participant characteristics (Gale et al., 2013). Analysis was conducted according to the five stages outlined by Ritchie and colleagues (Ritchie, Lewis, Nicholls, & Ormston, 2013):

1. *Familiarization with the data:* RL-P conducted all interviews, examined each transcript for accuracy, and read each transcript several times.
2. *Creating an initial thematic framework*: RL-P, PB, and NR devised a preliminary framework based on independent analysis of three transcripts (15%). Data were independently organized according to concepts, themes, and sub-themes. Different data interpretations were discussed until agreement was reached. Two more transcripts were subsequently independently analyzed by RL-P, PB, and NR to further confirm the coding scheme.
3. *Indexing:* RL-P coded all transcripts according to the scheme, with new themes and revisions discussed with PB and NR.
4. *Charting:* Themes and supporting quotes were transferred to a Framework matrix, managed using MS Word.
5. *Mapping and interpretation:* The framework was examined within and across themes and participants to identify themes, subthemes, patterns, and relationships. To ensure the themes were grounded in participants’ accounts, illustrative quotations were used to characterize each theme and subtheme.

Higher order themes and sub-themes were initially established through an inductive (data driven) approach. Six initial themes were identified *((1) living with symptoms and side effects; (2) hope and struggle; (3) finding meaning and coping in the face of lung cancer; (4) interacting with the health system during survivorship; (5) work and financial issues; (6) connection and loneliness: navigating relationships as a lung cancer survivor*) and were subsequently compared to conceptual frameworks of living with cancer [16, 19]. Roberts et al. [23]’s revision of the Folkman and Greer [8] theoretical model of appraisal and coping closely aligned with study findings, particularly initial themes 1, 2, and 3. The six initial themes were, therefore, divided across two papers, with the current paper aiming to describe survivors’ inner psychological experiences of living with lung cancer with a focus on appraisal and coping, and the second paper focusing on external life domains impacted by lung cancer (work, finances, relationships) and ongoing healthcare. Roberts’ model was utilized to inform the presentation and interpretation of study results, with minor amendments made to theme titles to better align with the model.

## Results

### Quantitative

Twenty individuals (10 female) participated in the study, with mean age 69 years (range 30–84 years). Of the six people diagnosed with stage I/II lung cancer, two reported subsequent disease progression. Participants were on average 17 months (range 10–24 months) post-initial lung cancer diagnosis. The majority had received a novel therapy such as IO (*n* = 10) or TT (*n* = 4), usually in combination with other treatments. Most participants (*n* = 14) were receiving medical management for two or more other health conditions or risk factors, with the most common being high blood pressure (*n* = 7), high cholesterol (*n* = 6), and heart disease (*n* = 5). Global QoL scores ranged from 33 to 94 (mean = 72, SD = 14), while distress scores ranged 0–10 (mean = 3, SD = 3). See Table 1 for participant demographic factors, clinical data, and PROs.

**Table 1:** Participant demographics, clinical data, and PROs (*N* = 20)

	Total (%)
Demographics	
Gender male	10 (50%)
Age range (mean) in years	30–84 (69)
Education	
Intermediate certificate/year 10	6 (30%)
Leaving certificate/year 12	3 (15%)
Technical certificate/diploma	4 (20%)
University degree	6 (30%)
Not reported	1 (5%)
Employment status	
Currently working	8 (40%)
On leave	1 (5%)
Retired	11 (55%)
Marital status	
Married/living with partner	10 (50%)
Widowed	5 (25%)
Single (never married)	3 (15%)
Divorced	2 (10%)
Country of Birth	
Australia/New Zealand	15 (75%)
Europe	3 (15%)
United Kingdom	2 (10%)
Location of residence *	
Metropolitan	14 (70%)
Rural	6 (30%)
Smoking status	
Previously smoked	14 (70%)
Never smoked	6 (30%)
Clinical data	
Stage at first diagnosis	
I	2 (10%)
II	4 (20%)
III	8 (40%)
IV	6 (30%)
Time since diagnosis range (mean) in months	10–24 (17)
Histological type	
Non-small cell lung cancer	18 (90%)
Small cell lung cancer	2 (10%)
Current disease status	
Early stage, no evidence of recurrence	3 (15%)
Recurrent	2 (10%)
Locally advanced or metastatic	15 (75%)
Treatments received #	
Lung resection	8 (40%)
Chemotherapy	10 (50%)
Radiotherapy	11 (55%)
Immunotherapy (IO)	10 (50%)
Targeted therapy (TT)	4 (20%)
Comorbidities ^	
0 other chronic health conditions	3 (15%)
1 other chronic health conditions	3 (15%)
2 other chronic health conditions	10 (50%)
3 + other chronic health conditions	4 (20%)
PROs	
Quality of Life EORTC-QLQ-C30 range (mean, SD)	33–94 (72, 14)
Distress NCCN Distress Thermometer range (mean, SD)	0–10 (3, 3)

^*^Based on postcode of residence

^#^Most participants have received more than one treatment

^Other chronic health conditions such as cardiovascular disease, arthritis, obesity, neurological disorders, endocrine disorders

### Qualitative

**Fig. 1:**
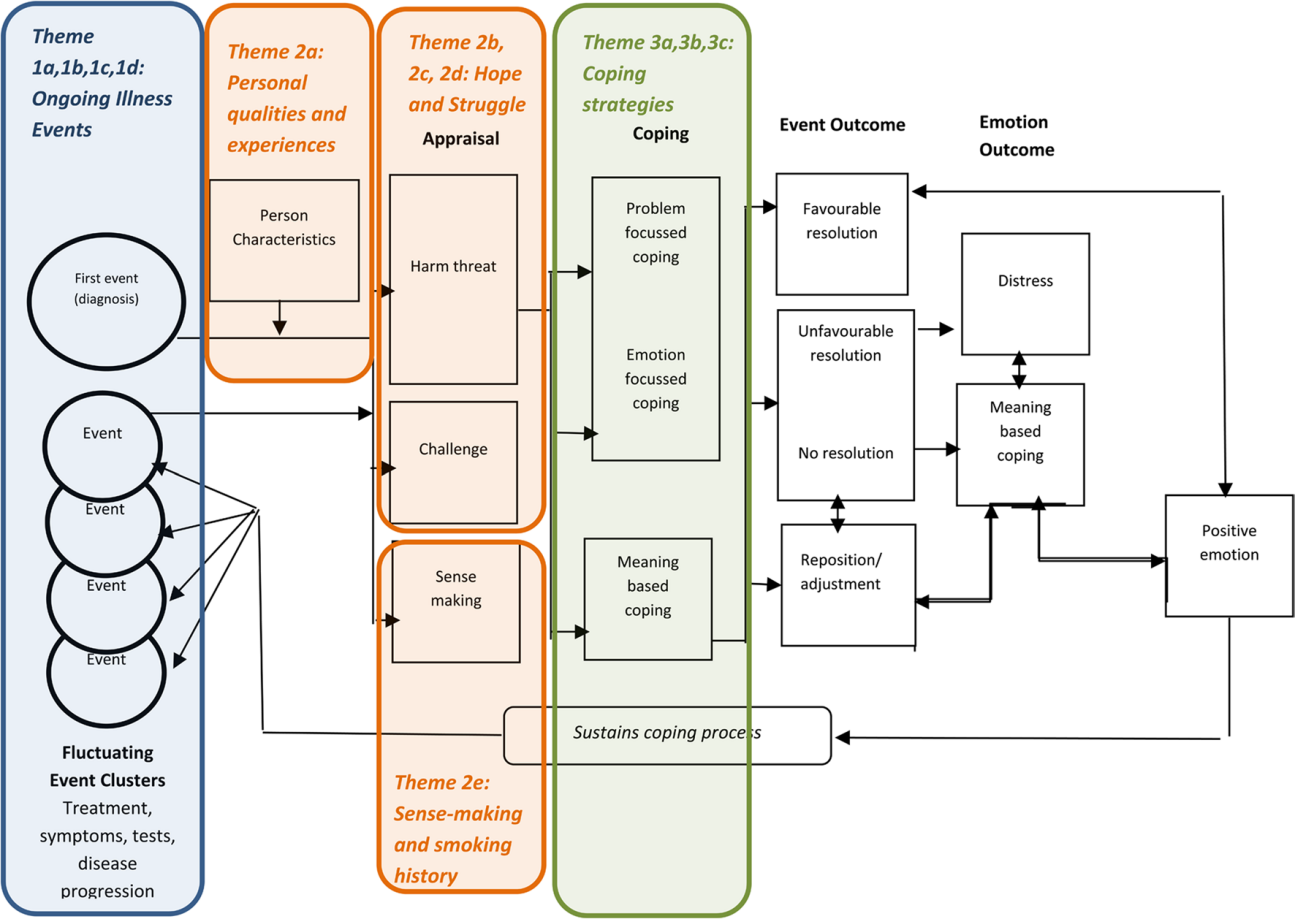
Mapping of current study themes to Roberts et al’s [23] advanced cancer adaptation of Folkman and Greer Coping and Appraisal Model

Three overarching themes related to survivors’ inner psychological experiences of living with lung cancer were identified: (1) *Ongoing illness events: experiencing the challenges of lung cancer, treatment side effects, and medical complexity;* (2) *The dichotomy of hope and struggle: adjusting to life with lung cancer*; and (3) *Coping strategies: learning to live with lung cancer*. Themes and subthemes, and how they map to the Roberts [23]’ model are depicted in Fig. 1. See Tables 2, 3, and 4 for additional illustrative quotations.

**Table 2:** Additional participant quotes for Theme 1: Ongoing illness events: experiencing the challenges of lung cancer, treatment side effects, and medical complexity

Theme 1a: Treatment and recovery	“After surgery, you have the feeling that you have been run over by a truck. I needed to recover, regain a bit of strength” [Male, IO, 50–59] “I got through [surgery]…. It was tough but I got through it. And I went to rehab and gradually increased my respiratory capacity. And I was doing fine living a relatively normal life… Then I didn’t get better as much as I expected to. [Later in the year] I had a chest infection and an X-ray, showed two metastases. [Doctor advised] we’d do radiotherapy, a second time. So I had a second lot of radiotherapy. And the scan shows [tumour reduction]. So the radiotherapy did have an effect. And now I’m on immunotherapy.” [Male, IO, 70–79] “My life so far has not been, greatly impacted by the cancer, you know I’ve managed to deal with it, cope with it psychologically and everything else. And physically, I haven't had great suffering [Male, IO, 70–79] “No physical side-effects. No. I didn’t lose my hair. I questioned the doctor at one stage if they had the right stuff. They gave me a box of pills for nausea and I never used one” [Male, IO, 70–79] “The cancer hasn’t impacted that much on my life…. yet” [Male, IO, 70–79]
Theme 1b: Lasting physical effects	With the nausea it's very hard to go out to lunch and dinner… The appetite is not there… I've lost a bit of weight [Female, IO, 70–79] “Since the radiation, I can’t taste too much, which is the reason why I don’t eat a lot because the food's tasteless. It's like chewing straw” [Male, TT, 80–89] “Breathlessness has been one of my downfalls…. it’s got much worse now” [Male, TT, 80–89] “Fatigue, definitely. Sometimes often I need to just lay down for half an hour in the afternoon which I've never done before” [Female, IO, 70–79]
Theme 1c: Navigating the uncertainty of new therapies	“It [immunotherapy] is building up the body to fight rather than injecting it with something foreign… Even though I knew nothing about it, I thought boosting the immune system so that it can fight whatever foreign body comes in and learns to fight that means we are strengthening. To me it was a more biological, reasonable thing to use part of your body to fight an alien” [Female, IO, 70–79] “I'd heard all of the side-effects like the nausea, the skin, the mouth ulcers, all these side-effects that were happening and I can remember saying to my husband, “This is a bit odd. Maybe these tablets aren’t working. I'm not getting any of the side-effects” [Female, TT, 70–79] We're not sure that’s [digestive issue] related to immunotherapy. It could be- it’s a very unknown disease. What the true causes are. I don't think it's actually related to my immunotherapy. I think it's just related to me being in my 50’s, it could have happened anyway” [Male, IO, 50–59] “I’ve got (skin rash). So next time I see the doctor I’ll just see if maybe I should be going back to a dermatologist because I’ve started to get a bit now on my side as well. I don't know if that's a side-effect from the immuno-stuff” [Male, IO, 70–79]
Theme 1d: Living with multiple chronic illnesses	I take a lot of tablets myself… five in the morning and I think there’s about six at night… so I have quite a few things wrong with me [Female, TT, 70–79] I don’t know that it’s so specifically related to lung cancer, as generally to my lung health. I still think the majority of my symptoms are because of my otherwise compromised lungs, because I keep getting these infections [Female, No IO/TT, 70–79] “I didn’t have chemotherapy to start with, because I have a neurological problem, and my neurologist… thought that perhaps it would be unwise to go into traditional chemotherapy because it's neurotoxic” [Male, IO, 70–79] “Breathlessness has been one of my downfalls. I’ve always been breathless. I’ve never been able to run on a sporting field and finish a game. Its got much worse now [since lung cancer diagnosis]. I take a puffer with me everywhere I go now” [Male, TT, 80–89]

**Table 3:** Additional participant quotes for Theme 2: The dichotomy of hope and struggle: appraising and adjusting to life with lung cancer

Theme 2a: Drawing on personal qualities and experiences	I’ve had this [separate illness] wrong with me and I’ve got through that. That’s been over many years. I know I’ve got this wrong [another illness] with me. Oh, that’s been many, many years. Oh, no, I’ve got lung cancer. OK. I’m sure I can fight this one too [Female, TT, 70–79] “My insurance company is [paying a living wage] so perversely, my cancer has maybe put me in a better [financial] position. Look, I guess it’s part of being lucky… I’m seeing more benefits than negativity at the moment.” [Male, IO, 50–59]
Theme 2b: Focusing on living, preparing for dying	“It’s silly to keep worrying, worrying, worrying about things- when you can’t actually do anything about it. You have to accept that that's what's happened to you. Just make the most of life” [Male, IO, 70–79] “Every time I talk about getting a will together, my wife is not that happy about that. But I say hey, this is a reality. At the moment things aren't clawing at the door so to speak, so I’m not giving it that much chance to be a great worry, but there are certainly things to put in place”[Male, early-stage, No IO/TT, 60–69] “There actually really is no use in worrying too much. It doesn’t achieve much. So you’ve just got to be cautious and keep an eye on things. I’ve already had the bonus of an extra year and six months of life already” [Male, IO, 50–59]
Theme 2c: Loss and fear—emotional struggles of living with lung cancer	“Some days I find I don’t handle it. I can have days where I just wander around the place. I don't know what to do with myself. I've had bouts of where I do have big cries. I struggle to get my breath with it” [Female, early-stage, No IO/TT, 50–59] “Like last week I had I had an enormous nose bleed and I thought here we got a, you know, I’ve got an aneurysm. You know, your brain jumps to conclusions” [Male, TT, 80–89] If you’re sitting waiting to die, you do get a bit anxious about what’s next [Male, TT, 80–89] The week before the scan I am a mess. A shocking, shaking mess. I don’t know how many years it takes off my life each time because I'm just thinking it's going to be a massive (cancer growth) there, every single time” [Female, IO, 70–79] “When I have tests coming up like I have now it gradually works its way to my forefront of mind. And so at the moment, with tests coming up later this week. And then the period between having the CT scan and seeing the oncologist is torture” [Male, IO, 60–69]
Theme 2d: Novel treatments as a beacon of hope	“I had three cycles and I had another scan. The tumor had shrunk by 80 percent. So now we’re talking instead of being the size of an apple, it’s now the size of a grape”- [Male, IO, 50–59] “As far as [doctors are] concerned, they’re pretty hopeful that they’ve pulled off a miracle” [Female, IO, 70–79] “[Immunotherapy] is stopping at the end of June, and I’ve already said to my oncologist, “there’s still something there, it’s probably scar tissue…But could we just go on a bit longer [with treatment]?” [Female, IO, 70–79] “And as far as immunotherapy goes, a number of the nurses have said to me, for the people that it works for, it’s a miracle drug. It’s just that at this point, they don't really have a way of predicting who it will work for” [Female, IO, 70–79] “I’ve read every Lancet article that exists on [specific immunotherapy drugs], immunotherapy, and the KEYNOTE-598 trials. I have read all of the results from Phase I, II… Well on the KEYNOTE 598 trial apparently, the results for most people on that trial are pretty bloody good” [Male, IO, 50–59]
Theme 2e: Smoking history and lung cancer—making sense of the diagnosis	“Yes, I pay the price for my indiscretions and my wild life and my smoking too much… It’s a fact of life. It's there. Can’t do anything about it. So we’re going to make the most of it and get on with the rest of life. You mustn’t let it make you feel depressed… You’ve got to face the problem and brooding too much on the possible cause and what if is a waste of time [Male, IO, 70–79] I shouldn’t have smoked, so what has happened is because of what I did… But it's a bit too late and I'm not going to dwell on that. It is what it is and I've got to get on with life and not think about it. [Female, No IO/TT, 60–69] Look, I was a smoker when I was 13. I used to [steal] cigarettes out of my mum's bag because… everybody smoked. That's what you did. And you were encouraged to smoke. We used to get cigarettes in the [Military] they gave you 200 a month. I was not aware [of the risk of smoking] at all. [Male, TT, 80–89] “I was a packet a day [smoker], so that’s not good. I know that's probably part of it. [Also worked in industry with high asbestos exposure]And in those days, asbestos was not taken that seriously. So it's probably with the smoking, but you can't pinpoint how it started. If I never would have smoked, it wouldn’t have happened maybe. It’s there, it’s done, there's nothing I can change about it. [Male, IO, 70–79]

**Table 4:** Additional participant quotes for Theme 3: Coping strategies: learning to live with and around lung cancer

Theme 3a: Emotion-focused coping	I don’t think along those lines [cancer progression] because if I did, I think that will be the worst thing in the world. It’s like a light switch and I’m not going to turn the lights off. Yes, [cancer progression] is in the back of your mind but– I often remind myself that I’m going to beat this so that is it, and I’m positive I really think I will. At the end of the day, I’ll go nuts if I don’t keep believing that [Female, No IO/TT, 60–69] “That night I was lying in bed elated that I had gotten on the [clinical] trial. And then I started feeling something in my spine… I was back in the hospital two days later with screaming agony because my sciatic nerve runs right through the cancer site in the spine. And an immune response had occurred, which had swollen that area and was crushing my sciatic nerve- the cells had suddenly switched on to say “oh that's cancer we’ve got to eat that”. So it’s just incredible. My treatment has been, there’s only one word for it, and that’s completely miraculous. So just basically the luckiest lung cancer patient around” [Male, IO, 50–59] “The first couple of nights, I didn’t sleep… then I realized I was looking too far ahead of myself. I went for a long walk and I decided I live by the day. And don’t look any further than tomorrow. And that’s when I started sleeping again” [Male, IO, 70–79]
Theme 3b: Problem-focused coping	“So I quite often ask to take my own big fat medical file down to the treatment area and while I am waiting I’ll quickly have a flick through just make sure that I'm across all of the paperwork. I’m heavily engaged. It's your life” [Male, IO, 50–59] “I'm actively trying to overcome the shortcoming of the lobectomy, I am conscious of it. A healthier lifestyle, exercise, no stress. I’ve cut out sugar, I’ve cut out red meat. I’ve cut out dairy. These are all things [book about beating cancer] makes clear might make a difference [Male, IO, 60–69] “It used to take over my life a lot more at the beginning when I’ve, just daily appointments, the phone ringing all the time, what have you. It actually took over my life in the beginning to the point where in the end I cancelled my mobile phone deliberately so that people couldn’t ring me from the hospital and stuff me up in ten minutes where I'd found something joyful to do” [Female, IO, 70–79] “I bought a bike and I’ve ridden 3000 kms on the bike. And that’s been a whole new discovery. I had to buy a special suspension seat so that my back [with spinal metastases] doesn’t get vibrated” [Male, IO, 50–59] “I can’t do as much because really as soon as I walk and have a hill at the side I really am out of breath. So playing golf now I can’t walk the course; I need to get a cart but at the same time I still play golf two, three times a week and very much enjoy it” [Female, IO, 70–79]
Theme 3c: Meaning-based coping	I think that the outcome of this as being positive and the realisation of, and appreciation for, life in general has put me in this position that I get less quickly irritated at work. I was usually one of those people that the moment that something went a certain way within the workspace, that I got annoyed by things I can now more easily now actually say, “OK, it is what it is”. I don’t take everything so seriously anymore [Male, IO, 50–59] I’m really proud of myself that after they tell me "you've got cancer" that is when your head spins, and I was looking way too far ahead, and I was thinking “alright… don’t worry about it. II said to myself, I'll get a stomach ulcer. So I was very impressed with myself that I could do that, not think ahead [Male, IO, 70–79] I wake up every morning and say, “thank you, God, for another day”. I literally do. I came quite quickly to realise that that you… Once you realised how precious life is and you don’t want to lose it. Remember for the time you have got how precious it is and make the most of it [Female, IO, 70–79]

#### Theme 1: Ongoing illness events: experiencing the challenges of lung cancer, treatment side effects, and medical complexity

Participants endured many illness *events* throughout their lung cancer experience. Most received multiple treatments and described a period of adjustment and recovery after each treatment. Some experienced ongoing effects of treatment. Many described the ongoing nature of IO/TT and the uncertainties related to receiving a novel treatment. Participants also described the how illness events from other comorbid illnesses complicated their experience of lung cancer. As depicted in Fig. 1, these multiple illness events can be compounding and fluctuating, and the nature and impact of these events are likely to influence an individual’s appraisal of their illness.

#### Theme 1a: Treatment and recovery

All participants described a “roller coaster” of illness events (e.g., tests, treatments) that had occurred since diagnosis. Many described a process of post-treatment recovery and adjustment (particularly after lung resection surgery and chemotherapy) and the relief of finishing treatments and returning to a sense of “normal.”

*It was such a relief to get off [treatment] and get life back to normal. There were lots of things that went on during [treatment] that weren’t great, radiation burned my oesophagus. All those things, they’re downers- but then you get over them and you’re encouraged- it’s a real seesaw and you have to trust… it is going to pass [Female, IO, 70-79] *

Some participants described additional complications, such as disease recurrence/metastasis, and subsequent treatments—often IO/TT. While these events were perceived as threatening, receiving a novel therapy such as IO/TT (with their positive reputation as being successful) encouraged and sustained some.

*As soon as I heard [doctor] say there’s this thing called immunotherapy, I rushed straight to [internet]… And 20 minutes of research told me that this was the answer. That switching your own body's immune system on to recognising the foreign body inside its body was the answer. I was keen as mustard to get it. [Male, IO, 50-59]*

For many, the physical effects of the illness and treatment were short-lived, manageable, and not as bad as expected, while others reported effects varied over time. Some felt lucky for not experiencing significant symptoms/side effects to date but acknowledged the likelihood of illness events to come. One participant noted “I presume that there will be some suffering to come in the future” *[Male, IO, 70–79].*

*I feel as though I’ve been – so far – very fortunate with it. I didn’t feel pain, the surgery, although it hurt, was manageable. Chemo, I’ve read the side-effects and I reckon I came out of that very well. [Male, early-stage, no IO/TT, 60-69]*

*It's been up and down… A couple of good days, four or five or six bad days. I feel very listless… I didn’t even feel like getting out of bed. But in recent weeks, I’m up and playing golf twice a week. [Male, TT, 80-89]*

#### Theme 1b: Lasting physical effects

Participants reported lasting physical effects of illness and treatment such as fatigue/lethargy, breathlessness, and coughing. Compounding fatigue was poor sleep, which some participants attributed to chest or back pain. Another ongoing issue for some was lack of appetite and reduced enjoyment of food, which for some was caused by nausea from IO/TT. While not experiencing specific ongoing effects, some acknowledged they felt older and lacked vitality. Most participants reported they were learning to adapt to these effects and some noted symptom improvements over time.

*When I try and lie on the left side, where the operation was, you get that constant throbbing... So I try and roll on the other side and you feel like your lung is hitting up against the heart. So some days I sort of twist from one side to another… I don’t sleep well. [Female, early-stage, no IO/TT, 50-59]*

#### Theme 1c: Navigating the uncertainty of new therapies

Half of participants were receiving/had received IO and four were currently on TT. Most participants described the long term or ongoing nature of these novel therapies, which may be received for 2–3 years (IO) or indefinitely (TT), and described the variety of *illness events* that occurred as a result of receiving them. Many noted the medications were “working well” to control the cancer, but acknowledged this could change in the future.

*The cancer at the moment is quite controlled and it will be quite controlled until … my body stops responding to the medication… my medication could stop doing well with me at any given time. [Female, TT, 30-39]*

Most were aware that IO/TT are newer classes of drugs with less long-term evidence, and acknowledged multiple treatment uncertainties, including how long the treatment would continue, how long it would be effective, and what the ongoing effects would be.

*So now what happens with this [IO], I asked my oncologist. She said after two years we take you off treatment and do three-monthly scans and hopefully by then the body will have learned to keep fighting, but of course, it is open to whatever might happen. I might have to go back on treatment... It’s very early in lung. Immunotherapy has been used for longer on skin cancer, but not with lung. When I started it had only been used for about a year. So this is all very exploratory [Female, IO, 70-79]*

Some participants viewed IO/TT as no different to chemotherapy, while others noted they were pleased to be receiving IO compared to chemotherapy. This was based on either their own experience of chemotherapy or its reputation as being unpleasant.

*[Doctor] highly recommended immunotherapy treatment. Which I had never heard of. I had heard of chemotherapy and that absolutely terrified me [Female, IO, 70-79]*

Most survivors reported tolerating IO/TT well. Some reported few/no side effects, and recalled wondering if the IO/TT was actually working. Several others experienced known side effects such as skin rashes, tiredness/fatigue, nausea, diarrhea, and joint stiffness, with most stating these were manageable. Of those who reported side effects, a few reported uncertainties about how side effects should be treated, by whom, whether they would resolve, and whether certain symptoms were the result of IO/TT, another health problem, or were part of normal ageing. A few participants reported experiencing unexpected issues such as brittle hair and nails, pins and needles, and digestive issues, and some were confused when their doctors stated that health issues (which occurred at the same time as commencing IO) were unrelated to treatment. This raised uncertainty about how to treat the issues and what their trajectory would be.

*I’m getting pins and needles in my right hand … I had x-rays taken and it didn’t show any injuries there, no arthritis, no gout… I spoke to the oncologist. He said as far as he was concerned it was nothing to do with the [IO] I've been having. [Male, IO, 70-79]*

Three participants experienced less common and serious side effects affecting the endocrine system, believed to be linked to IO. While all three expressed acceptance of these adverse events given the potential effectiveness of IO, they did acknowledge they are now on lifelong additional medications and live with the ongoing possibility of further medical complications.

*They discovered that [bodily system] had been destroyed. That was a direct consequence [of immunotherapy].… People need to be aware that this has occurred and may well occur again. [Female, IO, 70-79]*

#### Theme 1d: Living with multiple chronic illnesses

For some, lung cancer was one of several ongoing health issues that predated their cancer (e.g., chronic pain, heart disease, COPD) or appeared during—and possibly as a result of—treatment (e.g., thyroid issues). Participants described the complexity of living with lung cancer and other conditions, particularly related to treatment and polypharmacy. Two participants found the number of appointments and specialists to be overwhelming and distressing. For some, this medical complexity meant being unsure which illness or treatment was causing certain symptoms, and which health professional should manage them.

*There was just too much. With going to [vascular doctor], going to the eye specialist, and I had just been in hospital. There was too much for me to take in. And I just lost the plot…I thought oh no I’ve got four people to go and see now [Female, IO, 70-79]*

While most comorbidities were well managed, some participants reported compounding impacts from lung cancer and other conditions. This was particularly pronounced for those with comorbid respiratory conditions, as the lung cancer and treatments appeared to worsen their (already poor) respiratory function.

*I’ve had back surgery [for a pre-existing injury]. But because of that injury, it doesn’t help with the breath[lessness] part as well. Trying to work in the garden, the back aches and things throb. And I don’t know whether it is the back, the lungs or both. [Female, early-stage, no IO/TT, 50-59]*

#### Theme 2: The dichotomy of hope and struggle: appraising and adjusting to life with lung cancer

Most participants were diagnosed with advanced lung cancer, or subsequently experienced disease progression. While aware of the seriousness of their diagnosis, most participants said they were “hanging in,” their cancer was stable and “under control,” and expressed optimism. The few who received treatment with curative intent for early-stage cancer felt they had “dodged a bullet.”

As depicted in Fig. 1, many participants appeared to draw on *personal characteristics*, such as individual qualities and experiences as they adjusted to life with lung cancer. These personal characteristics may influence how a person appraises their disease. Indeed, participants’ appraisals of their lung cancer experience varied. While many described emotional struggle related to fear of disease recurrence/progression and death, they also highlighted positive emotions such as gratitude for extra time to enjoy life and hope for the future. These themes were particularly strong among those receiving IO/TT.

#### Theme 2a. Drawing on personal qualities and experiences

Participants described personal characteristics which they believed helped them to live with lung cancer. Some described qualities such as “resilience,” “pragmatism,” and “self-discipline” which they believed helped them to face, and live with, the cancer. A few believed that they possess unique skills which help them adjust, like an ability to see humor in challenging situations or being able to find joy in the present moment.

*I think I’ve got, almost an automatic mindfulness system of my own that I’ve always used, before people started to write books about it… Most of the good outcomes from a mental perspective in relation to my illness probably come from an already present resilience. [Male, IO, 50-59]*

Related to existing strength and resilience, many participants noted they had already endured exceedingly difficult experiences in their lives—prior illnesses, deaths of family, and divorce. They viewed lung cancer as another of life’s challenges, found strength in their ability to survive previous challenges, and drew on similar internal resources to adapt.

*During my life, I’ve been through quite a lot. And that probably also helped in building up that resilience. Many people who know me said to me, “what you’ve been through could have knocked you to the dark side”. At some point you make decisions in your life, what you do with what you experience. Does it get you down, or does it motivate you to do better? [Male, IO, 50-59]*

Many participants identified as “positive” or “lucky,” and appeared to focus on the good aspects of their lives, and the situation they were in. Even when bad things were happening for these participants, their hope and endurance were sustained through a focus on a positive mindset and the ways they were fortunate.

*I’m a person that feels very positive and I’m certainly not going to sit in the corner and wait for it to finish me off. [Female, IO, 70-79]*

#### Theme 2b: Focusing on living, preparing for dying

The majority of participants appeared to approach their cancer with an understanding of the *threat* of their diagnosis/prognosis, the prospect that death may be an outcome of their cancer, and the need to “get affairs in order.” Many acknowledged they think about death, particularly practicalities such as preparing wills and passing on heirlooms. However, most tempered these thoughts with a motivation to live well, to not worry about things they cannot control, and a commitment to “make the most of every day.” Many participants were able to move from seeing the cancer as a *threat,* to viewing it as a *challenge,* that can be learned from or can result in positive changes to one’s life. Some participants appeared to have reached a level of acceptance about their situation and reported gratitude for extra time, particularly those on novel therapies who had lived longer than initially anticipated.

*You read the death notices and they say so-and-so died after a long battle with cancer. And I don’t actually see it as a battle. The word battle to me brings up strife… and fretting. To me, it’s about developing more peace within myself, even though I’ve given myself all of these tasks to do around the house, every time I get one done, like writing this journal, I feel good about that.... Kind of tying up my life, and I needed a jolt into doing that [Female, IO, 70-79]*.

For many participants, *threat* and *challenge* were not mutually exclusive, rather demonstrating an ebbing and flowing between these appraisals as the disease and its impacts fluctuated over time.

#### Theme 2c: Loss and fear- emotional struggles of living with lung cancer

While most appeared to be adjusting to the reality of the lung cancer while focusing on hope or acceptance, a few participants described more negative emotions and experiences, appearing to struggle to adjust to their diagnosis, prognosis, and the ongoing effects of lung cancer. A common characteristic of these participants was a focus on *loss—*of capability, of their pre-cancer life, and of a future. For some, this had existential roots, while others mourned losses caused by lung cancer and its sequelae, such as feeling useless and powerless in their lives due to fatigue, social isolation, lost feelings of competence, or a lack of direction.

Additionally, one participant described herself as “not able to handle it,” demonstrating a lack of self-confidence in her ability to adjust and cope. A couple of participants disclosed they sometimes questioned whether life was worth living.

*I do see a counsellor for depression because I get very frustrated because I used to be a very active fit person and it’s just like one thing after another is tumbling down on me at the moment [Female, early-stage, no IO/TT, 50-59]*

*So there was a point where I felt really helpless, like I didn't do anything with my life. I’m just staying in my room all the time, not doing anything [Female, TT, 30-39]*

Several participants, even those who appeared to be adjusting well, reported occasional anxiety and fear of cancer recurrence/progression “sneaking” into their awareness, particularly if they had symptoms or during quiet times when they had no distractions. For some, the time leading up to a scan and while waiting for results was the worst, where uncertainty and fears became more prominent and “consuming.” This highlights the ongoing illness *events* that people with lung cancer face, and the constant process of adjusting to threats.

*If I’m honest, there’s a worry lurking in the back of my mind that it might not be OK… Any time I cough I think “Oh Christ I hope there’s nothing wrong with my lungs” [Male, early-stage, no IO/TT, 80-89]*

#### Theme 2d: Novel treatments as a beacon of hope

While being treated with a novel therapy (IO/TT) constituted a series of significant *illness events* for many participants (see Theme 1c), receipt of these treatments—and particularly participants’ attitudes towards these treatments—also appears to have influenced their appraisal and adjustment to the lung cancer. Those receiving IO/TT reported living with uncertainty about their illness, acknowledged the cancer could recur or progress in the future, and some were aware of the limited evidence on long-term effectiveness of IO/TT. Despite this, the majority appeared to appraise this treatment and its relatively unknown long-term effectiveness as an *opportunity* rather than as a *threat.*

Most participants who had been treated with IO/TT expressed gratitude for this “miracle drug,” which symbolized a beacon of hope. This hope was influenced by their own positive disease response as well as, for some, keeping informed via news/journal articles on the rapidly changing landscape of lung cancer treatments.

A couple of participants noted the significant financial cost of IO and only recent Australian universal healthcare coverage of these therapies, which made them feel particularly “lucky” and grateful.

*Cure and cancer- those 2 c words never go together. And they're still not quite together yet. But… I was on three months to live and if I survive- that’s the cure for cancer. The only thing I’ve had is immunotherapy [Male, IO, 50-59]*

*I am very fortunate that I am able to do this on my pension. I’m on my knees every day with gratitude and thanks that I am having this immunotherapy treatment… because I couldn’t afford this in any other country. It is extremely expensive treatment. [Female, IO, 70-79]*

Given the hope surrounding novel therapies, several participants were anxious about treatment ending, wanting to remain on treatment for as long as possible. Some felt fortunate to be on a clinical trial, which made them eligible to receive treatment for longer than standard care. One participant was distressed when she had to cease IO due to health concerns.

The hope of cure or “extra time” that IO gave made some participants tolerate even severe side effects. One participant noted the lack of other options meant they felt it was “immunotherapy or death.” This person who had severe complications from IO said: “Honestly… I'm lucky to be able to have it [significant side-effects of immunotherapy] frankly. Plenty of people I know get a cancer diagnosis and they are gone in 3 months” [Male, IO, 50–59].

IO also gave some participants a sense of control; they felt they were “doing something” to fight the cancer. It also helped them to maintain contact with clinical staff, which they appreciated from medical and emotional perspectives.

*It’s a little bit frightening to me that in a few weeks’ time [after finishing immunotherapy] I won't be doing anything. I've lost my feeling that I'm treating it. Since I am quite a control freak, it's the sense of control that you're doing something [Female, IO, 70-79]*

Finally, a few participants who had early-stage lung cancer and had not received IO were reassured it was an option for future treatment, in the event of a recurrence.

*I wasn’t offered [immunotherapy] but – that’s in the back of my mind. Cross fingers, touch wood, at this stage it's OK. But [immunotherapy] will be the next thing I would go for- I've already looked into that [Female, No IO/TT, 60-69].*

#### Theme 2e: Smoking history and lung cancer—making sense of the diagnosis

As depicted in Fig. 1, how an individual makes sense of their illness can also impact upon appraisal and coping. Participants reflected on *why* they were having to deal with lung cancer, demonstrating a process of sense-making in their illness appraisal. For many, this was directly related to a personal history of smoking. While the majority of participants identified as previous smokers, the way they *made sense* of the lung cancer differed. Some took direct responsibility, while others appeared to reject the notion that their smoking history was solely to blame. Those with no history of smoking appeared to have a more complicated sense-making process. Interestingly, most participants reported that they experienced little internalized or perceived stigma from health professionals, family, or friends about their lung cancer, with many stating that they talked openly about their diagnosis.

Some acknowledged their lung cancer was likely a direct result of heavy smoking and therefore must live with the consequences of their actions. A few reported deep regret at smoking, while others appeared determined not to punish themselves or dwell on the past. Some appeared to channel their regret into advocating for others to quit smoking, using their own experience was a warning.

*I smoked like a chimney for 20 years, I rolled the dice and I lost. I grew up in the period where smoking causes lung cancer. That was that was common knowledge throughout my entire smoking life [Male, IO, 50-59]*

*I used to like my smokes. Now I see somebody with a smoke in their mouth and I think how stupid you are. You’re just playing with death because sooner or later it will get you. I’m an idiot for smoking. [Male, No IO/TT, 70-79]*

A few participants who had quit smoking decades before diagnosis felt “annoyed” to be “paying a price” for something they did so long ago, particularly given some had started smoking before the association with lung cancer was well understood.

*[I feel] fairly annoyed, I gave up smoking 40+ years ago. Like I shouldn’t be having to pay for that. However, I fall behind the fact that when I was 17 doctors would recommend you take up smoking to make you relax. It was a very different time. I’m just annoyed. I don’t feel guilty. I don't say “Oh I wish I hadn't smoked”. There’s no point in doing that. [Female, early-stage, no IO/TT, 70-79]*

Some previous smokers felt that while their smoking likely *contributed,* there were also other factors which may have caused the illness.

*I don’t know how I got it. It may well be smoking, but it’s not necessarily. It could well have been brought on by living in a city. [Male, early-stage, no IO/TT, 60-69]*

A few participants, mostly women, had never smoked and appeared to struggle to make sense of the diagnosis. Some felt confused as to *why* they had developed lung cancer, some felt it was unfair as they had done “all the right things” to stay healthy. A few took solace in knowing they were not alone when learning about the proportion of never-smokers being diagnosed with lung cancer. Some reported being quick to clarify to others that they had never smoked when discussing their diagnosis.

*I was [young], I feel like I had done everything right. I don’t smoke. I exercise frequently. I go hiking. I did rowing, boxing, tennis. And I worked with kids with disabilities, so I was active all day. I didn’t drink either” [Female, TT, 30-39]*

*I think in conversations about the [lung cancer diagnosis] I throw in the non-smoking quite quickly… They find it remarkable. In many cases people do think, it’s almost automatic, that that you were a smoker. [Male, IO, 50-59]*

#### Theme 3: Coping strategies: learning to live with lung cancer

Despite living with significant (and, for many, incurable) disease, most participants appeared to be coping relatively well. Many had found ways to process their illness, drawing on strategies to find peace and happiness. As demonstrated in Fig. 1, coping with illness is not a one-way process, but rather a continual cyclical process of changing illness events, evolving appraisals, and drawing upon personal resources and varied coping strategies.

#### Theme 3a: Emotion-focused coping

Many participants described emotion-focused strategies to cope with ongoing negative physical and psychological effects, particularly regarding fear of cancer progression. Many reported positive expectancies/optimism, such as trying to “look on the bright side.” Some compared themselves with others less fortunate, which helped gain perspective and gratitude. Several participants described engaging in positive reappraisal of difficult situations. One participant reframed a misdiagnosis as an opportunity for the healthcare team to put more effort into their ongoing care. Another described having to travel to a major city for IO resulted in being able to visit adult children more regularly.

*I'm in my 70’s doing well. I've been very fortunate. Opposite me [in chemotherapy suite] is a 23-year-old with a one year old baby who has got a serious cancer and a bald head”. [Female, IO, 70-79]*

A few participants described finding happiness by engaging in activities as a source of enjoyment beyond cancer. This included booking holidays, planning social occasions with friends, and eating enjoyable food. Related to this was engaging in activities as a form of distraction from thinking about the cancer.

*I just try and forget about it [thoughts about cancer recurrence]. Go and exercise, go and have dinner, drink red wine. I think part of it is forward planning. We’re kind of constantly booking something good to do, having stuff to look forward to. [Male, IO, 60-69]*

Some participants focused on living in the present. This helped avoid “what if” thoughts, instead enjoying moments of happiness. Similarly, some noted the importance of “compartmentalizing” the cancer.

*I just take it day by day. My future is putting my name down for golf on Friday and having a bet on the horses at the weekend. [Male, TT, 80-89]*

#### Theme 3b: Problem-focused coping

Some participants found proactively taking control over elements of their illness helped their coping. For a few, this was through being informed, engaged patients, and managing their own illness, where the feeling of taking action “into our own hands” and “doing something… not sitting around waiting” was perceived as psychologically beneficial. Some described focusing on their health and well-being, including diet and exercise, as a way to feel they were doing something.

*Initially it was scary... that changed when we got on the front foot and realised we needed to take aspects of this into our own hands, rather than just sit around doing what the Australian medical industry tells you to do. It helps mentally to feel like you are doing something. [Male, IO, 60-69]*

Some participants described engaging in problem-solving when faced with specific challenges. This was particularly prevalent among those living with ongoing physical effects of the cancer/treatment, such as breathlessness, lethargy, or pain. They described finding workarounds or alternatives so that they could still complete daily tasks and engage in activities.

*Getting the ironing board out of the cupboard and bringing it through is quite an effort now… So I got one with wheels on it. [Male, TT, 80-89]*

#### Theme 3c: Meaning-based coping

Some participants described changes in themselves and their perspectives. For a few, lung cancer resulted in realigned priorities and the gaining of perspective; for example, material possessions were deemed less important, or careers not taken as seriously.

*Realising that some things really aren’t important. Like if my husband drops a plate and breaks it, which happens quite regularly- what the heck?.. So getting my priorities a bit more in order that some things don't matter. You can't take anything with you [when you die]. [Female, IO, 70-79]*

A couple of participants described a process of re-defining themselves because of the loss of their careers or physical fitness. This included engaging in new hobbies to feel productive.

*I haven’t worked since that since the diagnosis. [My Job] was fun. I’ve actually been trying to [engage in construction hobby]… that has been my sanity project. I try to keep myself as busy as I can. Not being able to work is a real challenge, when it is your lifestyle as well as your work [Male, IO, 50-59]*

Several participants described personal growth since their diagnosis. For some, their ability to cope was a source of personal pride, others described a change in their appreciation of life and family.

*“I probably take in the moments of parenting more consciously… I’m very conscious of little moments of happiness and spending time with the [children]” [Male, IO, 50-59]*

Several participants found meaning, peace and/or hope by engaging with their spirituality and placing their future with a higher power. Some found comfort from their church community.

*There’s nothing in the end that you can do other than get some peace and put it in the hands of the experts. And that’s not only the medical people, but for me, that’s also putting it in the hands of a higher power [Female, IO, 70-79]*

## Discussion

Improvements in treatment and survival have rapidly changed the landscape of lung cancer [31]. This study is one of the first to describe the psychological experience of lung cancer survivors within the current era of novel immunotherapy and targeted therapies, and investigates their illness appraisal, adjustment, and coping strategies. Most participants in this study appeared to be adjusting to life with lung cancer. The majority considered most of the physical (e.g., fatigue, breathlessness, nausea) and psychological (e.g., uncertainty, fear of recurrence/progression) impacts to be manageable, and drew on personal qualities and strategies to cope.

This study directly contributes evidence to the validity of Roberts et al. [23]’s adaptation of the Folkman and Greer [8] model among those with advanced cancer, particularly the development and refinement of strategies to live with repeated, fluctuating, and ongoing *illness events*. In alignment with the Roberts et al. [23]’s model, this study found that participants draw on *personal characteristics* as they adjust to life with lung cancer. Many were able to engage with lung cancer as a *challenge* to learn and grow from rather than as a *threat* resulting only in loss. However, one facet of participant experiences that was not well captured within the Roberts et al. [23]’s adaptation was the level of *uncertainty* participants live with. For many, particularly those on IO/TT where evidence on long-term survival is lacking, they must live without clarity on an *event outcome*. Additionally, during the process of mapping initial themes to the model, it was apparent that the model does not describe the bidirectional influence of advanced cancer on relationships with family/friends, or how impacts on life domains such as employment and finances can influence coping and adjustment. These were significant themes highlighted by participants in our study, but which will be reported separately. Future versions of this model should consider how the experience of uncertainty (particularly among those receiving novel and emerging therapies) and how the broader sociocultural context of the survivor can be captured.

The majority of participants in our study were living with advanced, incurable lung cancer; however, most considered their cancer to be stable or “under control.” These individuals represent an emerging subset of cancer survivors living with lung cancer as a chronic illness [31]. A recent qualitative study of people living with protracted incurable breast and lung cancer found that as patients grew accustomed to having *chronic cancer*, the distress associated with the fluctuations of the cancer lessened, and they were able to remain hopeful and optimistic for the future [3]. These findings support the concept of *ongoing illness events* found in the current study and Roberts et al. [23]’s framework, where individuals learn to adapt to ongoing cancer events (treatments, tests, recurrence) and gain confidence in their ability to cope with each experience. Findings also support other recent studies [20, 31] which discuss the “duality” of living with lung cancer, whereby hope and positivity simultaneously exist alongside worry and struggle. Lung cancer survivorship care needs to be tailored to the unique psychological and existential needs of these populations living with chronic advanced cancer.

The optimism expressed by many participants is likely related to their beliefs about, and experiences of, newly available treatments IO and TT. Many participants viewed these treatments as a “miracle” and “game changer” and were buoyed by success stories they heard from health professionals or on the internet. Participants were aware of the uncertainty of if/how long the treatment would be successful for; however, this uncertainty appears to be perceived as an *opportunity* rather than *danger*, providing hope for the future. While novel treatments provide much needed hope and optimism for a longer life, clinicians need to be cognizant of balancing potential hype with reality when informing patients about IO/TT. Similar findings have been demonstrated among advanced melanoma patients receiving IO, where considerations of the trade-off between efficacy and toxicity may be disproportionately influenced by hype and hope [35]. Indeed, some participants in our study endured serious side effects of immunotherapy, others wanted to stay on treatment longer than recommended, and one participant who had to cease IO was highly distressed. Studies have also found that lung cancer patients on IO heavily emphasize the importance of survival over treatment burden [17]. Clinicians need to be aware of patients’ drive for hope and optimistic attitudes towards novel therapies when navigating treatment discussions, and provide clear and realistic information about efficacy and possible side-effects to facilitate informed decision-making.

Participants on IO/TT also described unmet needs regarding the recognition and appropriate management of possible treatment side effects. Some experienced known side effects of IO/TT (e.g., nausea, diarrhea, lethargy, joint pain), while others experienced unanticipated or unexplained issues which appeared alongside treatment (e.g., pins and needles, gastric complications). There was confusion about whether the issues were the result of IO/TT (particularly given the high prevalence of multimorbidity) and who they should be managed by. For many, these issues remained untreated or unresolved. Previous research has found that lung cancer patients on IO frequently under-report ongoing issues to their medical team, often because they believe they are not severe enough to report, can be managed without assistance, or are unsure if experiences are actually side-effects [17]. Routine enquiry from treatment teams about patient symptoms is key, particularly given patient’s confusion about what constitutes side effects and potential for under-reporting. A possible avenue for future practice is routine assessment and monitoring of patient reported quality of life measures specific to immunotherapy, such as with the Functional Assessment of Cancer Therapy-Immune Checkpoint Modulator (FACT-ICM) [10]. Further research is needed to establish the utility of this measure in clinical practice, as well as measures to appropriately capture the needs of those on targeted therapies, where different side-effect profiles exist.

While most participants were living well, a small but important minority did not appear to be adjusting as effectively. These participants appeared to focus on the *losses* they had experienced and did not engage in as many coping strategies. For them, ongoing illness events appeared wearing and defeating, compared to others who gained confidence from successfully navigating each event. Previous research has found that appraisal and coping can influence lung cancer patient outcomes. One study found that those with a constructive approach (seeing disease as a challenge to be met, positive redefinition of oneself) had lower rates of anxiety and depression and higher quality of life, whereas those with destructive attitudes (resignation, helplessness, anxious preoccupation) exhibited higher symptom burden, and poorer quality of life and psychological wellbeing (Jankowska-Polańska, Polański, Chabowski, Rosińczuk, & Mazur, 2019). Clinicians should remain alert to patients exhibiting psychological distress as well as those who may more subtly demonstrate a lack of adequate coping strategies, and refer for psychosocial support. Future interventions designed to improve coping among lung cancer survivors may benefit from drawing on the varied strategies utilized by this study’s participants, as literature on coping strategies among those living with lung cancer remains scarce [11].

The majority of participants had a personal history of smoking, with most having quit smoking prior to diagnosis. Interestingly, most participants held internalized shame and stigma, and negative attitudes towards others, for *smoking* behavior*,* but that stigma did not appear to extend as strongly to the diagnosis of *lung cancer.* Many acknowledged that their smoking history likely contributed to their diagnosis, but they did not want to blame or shame themselves for having lung cancer. Those who had never smoked appeared to distance themselves from the behavior of *smoking,* rather than the lung cancer. Most participants reported that they did not experience significant lung cancer stigma from health professionals, friends, or family. Participants’ negative smoking attitudes may be reflective of the extensive denormalization of smoking among the Australian community more broadly [5], particularly given the public health interventions and legislative restrictions which have been introduced [9, 27]. Additionally, research shows that people with lung cancer who currently smoke experience higher perceived and internalized stigma than those who previously smoked but had since ceased and those who have never smoked [34]. Given that we did not have any currently smoking participants in our study, it may be that we did not capture the experiences of those facing the highest levels of stigma.

Important limitations of this study must be acknowledged. Firstly, most participants were functioning surprisingly well, and demonstrated relatively high QoL and low distress. It may be that we did not capture the views of those who were more acutely unwell or did not have the (physical or mental) energy to participate in a lengthy interview. Our sample was heavily biased towards Australian- or European-born participants, and therefore, the experiences of culturally and linguistically diverse individuals were not captured. Future research should further purposively sample individuals with higher levels of distress, lower quality of life, and those individuals who have culturally and linguistically diverse backgrounds, as these survivors may experience unique challenges. As data collection occurred during the COVID-19 pandemic, face-to-face interviews were not possible and telephone interviews were utilized. Telephone interviews can have limitations, as establishing rapport can be difficult and non-verbal cues are not captured [26]. The interviewer did, however, utilize strategies to build rapport, allow flow of conversation, and to collect para-verbal cues as outlined by Burke and Miller [4].

Despite these limitations, this study’s strengths lie in its deep exploration of the experiences of people living with lung cancer who have undergone different treatment pathways and were at different stages of survivorship. Indeed, we were able to capture and contrast the experiences of those who have and have not received IO/TT and linked this to theoretical constructs resulting in important and novel insights on unique experiences among these populations. Additionally, recruitment via the EnRICH cohort study resulted in participants from across the state of New South Wales, meaning that this study was able to capture a broader representation of lung cancer survivors, with experiences not limited to participants from a single treatment center, and with wide variation in participants’ age, education level, and geographic location.

In conclusion, this study provides rich insights into the adjustment and coping of those living with lung cancer, with a particular focus on the experiences of those on novel treatments. Results demonstrate that overall, most individuals are living well with their ongoing illness, adapting to issues as they arise, and maintaining a sense of hope and optimism for the future. Findings suggest people living with lung cancer as a chronic condition, particularly those on long-term therapies IO/TT, may have unique psychological and symptom management needs which require ongoing assessment and management.

## Supplementary Information

Below is the link to the electronic supplementary material.

Supplementary file1 (DOCX 19 KB)

Supplementary file2 (DOCX 26 KB)

## Data Availability

The data that support the findings of this study are available from the corresponding author upon reasonable request.
