# Supplementary material for: Application of a revised model for coping with advanced cancer to qualitatively explore lung cancer survivors’ experiences of ongoing physical effects, novel treatments, uncertainty, and coping
Source: J Cancer Surviv. 2023 Jul 27;18(6):1754–70. doi: 10.1007/s11764-023-01417-x (PMC11502627; doi:10.1007/s11764-023-01417-x)
Supplement: Supplementary file 1 — Supplementary file1 (DOCX 19 KB) [file 11764_2023_1417_MOESM1_ESM.docx]

**SUPPLEMENTARY FILE A: Lung cancer survivor qualitative study- Interview guide**

*1. Diagnosis and treatment*

- Can you tell me about how you were first diagnosed with lung cancer?
- Can you tell me about what it was like during your active treatment of lung cancer?
  - *Have you been on, or offered, any immunotherapy or targeted treatment?*
- Can you tell me about what your ongoing care involves?

*2. Survivorship experience*

- Can you tell me what life has been like since completing your main lung cancer treatment?

*3. Survivorship challenges and coping strategies/support*

- What do you think have been the main challenges you have faced with regards to living with or beyond lung cancer?
- You mentioned your experience ___(side effects/symptoms/challenges/emotions)____, what helps you to get through that?
- How have you found follow-up care since you finished treatment?
- What is your sense of how you are managing life having been diagnosed with lung cancer?

*4. Psychological experiences /coping*

- How have you felt about yourself since you completed your main treatment for lung cancer?
- Do you ever have any worries about the cancer returning or getting worse?
- How do you feel about the future?
- Is there anything that helps you adapt to life with (and perhaps after) lung cancer?
- What gives your life meaning?
- Have you been part of any support groups or been offered any support services?
- Has the lung cancer impacted on your ability to do the things you like to do?
- Could you tell me about if/how your diagnosis and treatment of lung cancer has affected your ability to work?

*5. Family and community*

- Can you tell me about how your family/friends have been involved in your care since you completed your main lung cancer treatment?
- Can you tell me whether you feel you have experienced any stigma about having lung cancer?
- Do you feel that other people make assumptions about how you got lung cancer and what it has been like for you?
- *[For those with history of smoking]* Do you think your smoking history impacts on your lung cancer experience? If so, how?
- *[For those with NO history of smoking]* Do you think your history of not having smoked cigarettes impacts on your lung cancer experience?
- How comfortable do you feel talking to people about your lung cancer in general?

*6. Additional comments*

- Is there anything else you would like to add?
