# Supplementary material for: Application of a revised model for coping with advanced cancer to qualitatively explore lung cancer survivors’ experiences of ongoing physical effects, novel treatments, uncertainty, and coping
Source: J Cancer Surviv. 2023 Jul 27;18(6):1754–70. doi: 10.1007/s11764-023-01417-x (PMC11502627; doi:10.1007/s11764-023-01417-x)
Supplement: Supplementary file 2 — Supplementary file2 (DOCX 26 KB) [file 11764_2023_1417_MOESM2_ESM.docx]

**SUPPLEMENTARY FILE B.**

**Evaluating the current study against the Consolidated Criteria for Reporting Qualitative Research checklist (COREQ)**

| **Item** | | **Question/topic** | **Comment** |
| --- | --- | --- | --- |
| **Domain 1: Research team and reflexivity** | | | |
| **Personal Characteristics** | | | |
| 1 | | Interviewer/facilitator  Which author/s conducted the interview or focus group? | RL-P conducted all interviews (page 5) |
| 2 | | Credentials  What were the researcher’s credentials? E.g. PhD, MD | RL-P is a Psycho-Oncology Research Fellow Psychology, has a PhD, and is highly experienced in qualitative research (page 5) |
| 3 | | Occupation  What was their occupation at the time of the study? |  |
| 4 | | Gender  Was the researcher male or female? | RL-P is female (page 5) |
| 5 | | Experience and training  What experience or training did the researcher have? | - 1. RL-P has completed qualitative research training workshops, been mentored by qualitative experts, and has been a qualitative researcher for over 10 years. To date, she has published >15 peer reviewed qualitative studies. |
| **Relationship with participants** | | | |
| 6 | | Relationship established  Was a relationship established prior to study commencement? | Relationships were already established via the EnRICH Cohort Study (BB, KM), which participants were already involved in. After participants expressed interest in participating in this study to BB/KM, RL-P contacted participants to provide further information about the study and to obtain contact details to send the questionnaire to participants. After participants completed the questionnaire, the researcher contacted participants to schedule in a telephone interview. Rapport was built during these two points of contact. |
| 7 | | Participant knowledge of the interviewer What did the participants know about the researcher? e.g. personal goals, reasons for doing the research | Participants were informed that the study was a research study of living with lung cancer. They were told in general terms the importance of this research in understanding the experiences and needs of lung cancer survivors. |
| 8 | | Interviewer characteristics  What characteristics were reported about the interviewer/facilitator? e.g. Bias, assumptions, reasons and interests in the research topic | RL-P is a psycho-oncology researcher with an interest in understanding the needs of people living with and beyond cancer. |
| **Domain 2: study design** | | | |
| **Theoretical framework** | | | |
| 9 | | Methodological orientation and theory What methodological orientation was stated to underpin the study? e.g. grounded theory, discourse analysis, ethnography, phenomenology, content analysis | Thematic framework analysis (page 5), mapping to Roberts et al.’s (2017) Theoretical Model of Appraisal and Coping (page 5) |
| **Participant selection** | | | |
| 10 | | Sampling  How were participants selected? e.g. purposive, convenience, consecutive, snowball | Purposive sampling (page 4) |
| 11 | | Method of approach  How were participants approached? e.g. face-to-face, telephone, mail, email | EnRICH project officer contacted eligible participants by telephone and invited participation. Interested patients were subsequently contacted by the study manager (RL-P) by telephone to provide more detailed information. Information sheet and consent forms were provided by email or post, depending on the participants preference (page 4). |
| 12 | | Sample size How many participants were in the study? | N = 20 (page 5) |
| 13 | | Non-participation How many people refused to participate or dropped out? Reasons? | See Supplementary File C for number of people who dropped out and reasons why |
| **Setting** | | | |
| 14 | | Setting of data collection Where was the data collected? e.g. home, clinic, workplace | Telephone interviews were conducted from a private room, either in an office or home setting |
| 15 | | Presence of non-participants Was anyone else present besides the participants and researchers? | Non participation information was not collected. Three participants who initially provided consent had to withdraw due to significant illness. |
| 16 | | Description of sample What are the important characteristics of the sample? e.g. demographic data, date | See Participant demographics (pages 5-6 and Table 1) |
| **Data collection** | | |  |
| 17 | Interview guide Were questions, prompts, guides provided by the authors? Was it pilot tested? | | See Supplementary File A for interview questions. Interview questions were reviewed and refined by the authorship group. Further, the first three interview transcripts were read by NR and PB to ensure question phrasing and interviewer style were appropriate (page 5). |
| 18 | Repeat interviews Were repeat interviews carried out? If yes, how many? | | All interviews were completed in a single session with no repeat interviews |
| 19 | Audio/visual recording Did the research use audio or visual recording to collect the data | | All interviews were audio recorded (page 5) |
| 20 | Field notes Were field notes made during and/or after the interview or focus group? | | Post-interview reflection notes were documented immediately after each interview (page 5) |
| 21 | Duration What was the duration of the interviews or focus group? | | Interviews lasted on average 54 minutes, ranging from 27 to 97 minutes. (page 5) |
| 22 | Data saturation Was data saturation discussed? | | Recruitment continued until thematic saturation was reached (page 4) |
| 23 | Transcripts returned Were transcripts returned to participants for comment and/or correction? | | Transcripts were not returned to participants for comment or correction |
| **Domain 3: analysis and findings** | | | |
| **Data analysis** | | | |
| 24 | Number of data coders How many data coders coded the data? | | All interviews were coded by RL-P. |
| 25 | Description of the coding tree Did authors provide a description of the coding tree? | | Yes, see Results. |
| 26 | Derivation of themes Were themes identified in advance or derived from the data? | | Themes were derived from the data as per framework analysis (page 5). After initial analysis and thematic mapping, themes were subsequently compared to the Robert’s et al. (2017) Theoretical Model of Appraisal and Coping (page 5) |
| 27 | Software What software, if applicable, was used to manage the data? | | Microsoft Word and Excel (page 5) |
| 28 | Participant checking Did participants provide feedback on the findings? | | Participants were not asked to provide feedback on the findings. |
| **Reporting** | | | |
| 29 | Quotations presented Were participant quotations presented to illustrate the themes / findings?  Was each quotation identified? e.g. participant number | | Yes, see Results and Tables 2, 3, and 4 |
| 30 | Data and findings consistent Was there consistency between the data presented and the findings? | | Yes, see Results and Tables 2, 3, and 4 |
| 31 | Clarity of major themes Were major themes clearly presented in the findings? | | Yes, see Results and Tables 2, 3, and 4 |
| 32 | Clarity of minor themes Is there a description of diverse cases or discussion of minor themes? | | Yes, see Results and Tables 2, 3, and 4 |
